# Methylselenol Produced In Vivo from Methylseleninic Acid or Dimethyl Diselenide Induces Toxic Protein Aggregation in *Saccharomyces cerevisiae*

**DOI:** 10.3390/ijms22052241

**Published:** 2021-02-24

**Authors:** Marc Dauplais, Katarzyna Bierla, Coralie Maizeray, Roxane Lestini, Ryszard Lobinski, Pierre Plateau, Joanna Szpunar, Myriam Lazard

**Affiliations:** 1 Laboratoire de Biologie Structurale de la Cellule, BIOC, École Polytechnique, CNRS-UMR7654, IP Paris, 91128 Palaiseau CEDEX, France; marc.dauplais@polytechnique.edu (M.D.); maizeray.coralie@hotmail.fr (C.M.); plateau@bioc.polytechnique.fr (P.P.); 2 IPREM UMR5254, E2S UPPA, Institut des Sciences Analytiques et de Physico-Chimie Pour l’Environnement et les Matériaux, CNRS, Université de Pau et des Pays de l’Adour, Hélioparc, 64053 Pau, France; katarzynabierla@wp.pl (K.B.); Ryszard.Lobinski@univ-pau.fr (R.L.); joanna.szpunar@univ-pau.fr (J.S.); 3 Laboratoire d’Optique et Biosciences, École Polytechnique, CNRS UMR7645—INSERM U1182, IP Paris, 91128 Palaiseau CEDEX, France; roxane.lestini@polytechnique.edu; 4 Laboratory of Molecular Dietetics, I.M. Sechenov First Moscow State Medical University, 19048 Moscow, Russia; 5 Chair of Analytical Chemistry, Faculty of Chemistry, Warsaw University of Technology, Noakowskiego 3, 00-664 Warszawa, Poland

**Keywords:** methylselenol, diselenide, methylseleninic acid, thiol/disulfide exchange, redox equilibrium, *Saccharomyces cerevisiae* metabolism, toxicity, protein aggregation

## Abstract

Methylselenol (MeSeH) has been suggested to be a critical metabolite for anticancer activity of selenium, although the mechanisms underlying its activity remain to be fully established. The aim of this study was to identify metabolic pathways of MeSeH in *Saccharomyces cerevisiae* to decipher the mechanism of its toxicity. We first investigated in vitro the formation of MeSeH from methylseleninic acid (MSeA) or dimethyldiselenide. Determination of the equilibrium and rate constants of the reactions between glutathione (GSH) and these MeSeH precursors indicates that in the conditions that prevail in vivo, GSH can reduce the major part of MSeA or dimethyldiselenide into MeSeH. MeSeH can also be enzymatically produced by glutathione reductase or thioredoxin/thioredoxin reductase. Studies on the toxicity of MeSeH precursors (MSeA, dimethyldiselenide or a mixture of MSeA and GSH) in *S.* *cerevisiae* revealed that cytotoxicity and selenomethionine content were severely reduced in a *met17* mutant devoid of O-acetylhomoserine sulfhydrylase. This suggests conversion of MeSeH into selenomethionine by this enzyme. Protein aggregation was observed in wild-type but not in *met17* cells. Altogether, our findings support the view that MeSeH is toxic in *S. cerevisiae* because it is metabolized into selenomethionine which, in turn, induces toxic protein aggregation.

## 1. Introduction

Selenium (Se) is an essential trace element for mammals. It is involved in redox functions as the amino acid selenocysteine, translationally inserted in the active site of a few proteins [1]. Se has attracted considerable interest in the last decades for its reported beneficial effects on the prevention and/or treatment of several cancers and other diseases but also from a toxicological perspective because of the narrow margin between intakes that result in efficacy and those that are toxic [2].

Many studies have shown that Se compounds are selectively toxic to malignant cells compared to normal cells, highlighting their potential for cancer therapy [3,4]. There is also accumulating evidence that adverse health effects are associated with excess dietary Se supplementation [5,6]. Despite these studies, our understanding of the molecular mechanisms underlying Se toxicity remains limited. Metabolization of selenocompounds in vivo gives rise to multiple metabolites [7,8,9], the biological activity of which depends on their transformation into different active products [10]. Therefore, defining the metabolic pathways used by selenocompounds and characterizing the toxicity of Se metabolic intermediates are important steps to better understand both the beneficial and toxic mechanisms of Se in human biology.

Methylselenol (MeSeH, CH_3_SeH) has been suggested to be a critical Se-metabolite for anticancer activity [11]. Several studies have shown that Se-precursors metabolized into MeSeH are potent tumor inhibitors [12,13,14,15]. Methylseleninic acid (MSeA, CH_3_SeO_2_H) is an organoselenium derivative that can be converted into methylselenol by reducing agents. MSeA has shown encouraging anticancer activity in in vitro and in vivo models [16,17,18,19], although the underlying mechanism(s) of MSeA in anticancer activity remain elusive. Spallholz et al. proposed that MSeA triggered cell cycle arrest and apoptosis mediated by superoxide formation from redox cycling of MeSeH with oxygen and glutathione (GSH) [20,21]. However, MSeA was shown to induce apoptosis in prostate cancer cell lines without production of reactive oxygen species (ROS) [22]. Another possible mechanism accounting for MeSeH toxicity may be through its ability to reduce disulfide bonds of proteins and/or to form selenylsufide adducts with protein cysteine residues [23,24,25]. Because redox-sensitive cysteine residues play catalytic, structural, and regulatory roles in the biology of many proteins, reaction of MeSeH with these residues may impact many cellular processes including cell proliferation. For example, MSeA was shown to inactivate Protein kinase C, a receptor for tumor promoters, through a redox modification of critical cysteine sulfhydryls in the catalytic domain of the enzyme [26].

In vivo studies in the model *Saccharomyces cerevisiae* have significantly contributed to our understanding of the mechanisms that drive the mode of action and the toxicity of several Se compounds [27]. These studies have evidenced that the mode of action of Se is compound-specific. The inorganic Se compound sodium selenite (Na_2_SeO_3_) was shown to be reduced by intracellular thiols to the key metabolite selenide (H_2_Se/HSe^−^) that can redox cycle with oxygen and GSH resulting in redox imbalance and generation of ROS that kill cells mainly through DNA damage. Thus, deletion of several genes involved in DNA repair and the GSH redox pathway resulted in hypersensitivity to selenite/selenide [28]. Considering the importance of redox balance in cancer cells, this mechanism might contribute to explain the high sensitivity of tumor cells to selenite. In contrast, the toxicity of selenomethionine (SeMet) involves its metabolic conversion to selenocysteine and misincorporation of the latter in proteins resulting in protein misfolding and aggregation [29].

In this paper, we applied a strategy that gave insights into selenite and SeMet toxicity in yeast to identify metabolic pathways affected by MeSeH. We first investigated in vitro the enzymatic and non-enzymatic formation of MeSeH starting from MSeA, methylselenoglutathione (MeSeSG) or dimethyl diselenide (DMDSe). Then, we analyzed the toxicity of these MeSeH precursors in *S. cerevisiae*. Analysis of various mutant strains supports the view that MeSeH is converted into SeMet, leading to toxic protein aggregation in yeast cells.

## 2. Result and Discussion

### 2.1. Thiol Reduction of MSeA, MeSeSG, and DMDSe

It is generally accepted that MeSeH is produced spontaneously from MSeA by reaction with thiols such as glutathione (GSH) as schematized below (Equations (1)–(3)) [30].

(1) $$\mathrm{MeSeA}\;+\;2\;\mathrm{GSH}\;\to \;\mathrm{MSeOH}\;+\;\mathrm{GSSG}\;+\;H_{2}O$$

(2) $$\mathrm{MeSeOH}\;+\;\mathrm{GSH}\;\to \;\mathrm{MeSeSG}\;+\;H_{2}O$$

(3) MeSeSG + GSH ↔ MeSeH + GSSG 

                       

$$\mathrm{MSeA}\;+\;4\;\mathrm{GSH}\;\to \;\mathrm{MeSeH}\;+\;2\;\mathrm{GSSG}\;+\;2\;H_{2}O$$

**Figure 1:**
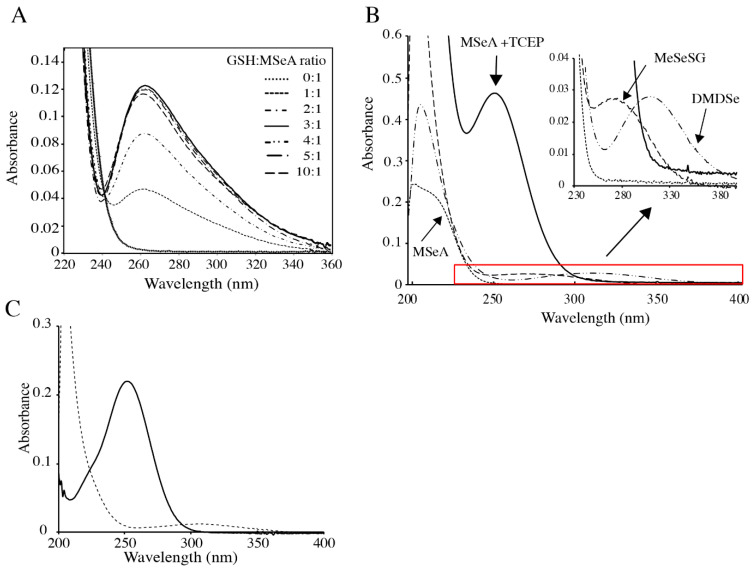
UV absorption spectra of various Se-containing species and effects of reducing agents. (**A**) Reduction of methylseleninic acid (MSeA) by glutathione (GSH). UV absorption spectra in 100 mM potassium phosphate, pH 7.4, of MSeA (250 µM) mixed with increasing amounts of GSH as indicated in the figure. (**B**) UV absorption spectra of various Se-containing species. The sample cuvette contained 100 µM MSeA in the absence (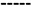) or presence (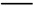) of 500 µM TCEP, 100 µM MeSeSG (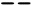), or 100 µM DMDSe (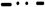) in 100 mM potassium phosphate, pH 7.4; spectra in the red box are extended in the inset. (**C**) Reaction of MeSeSG with methylselenol (MeSeH). MeSeH was produced by mixing 25 µM DMDSe with 25 µM TCEP in 100 mM potassium phosphate, pH 7.4. UV absorption spectra were recorded before (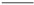) and after the addition of 50 µM MeSeSG (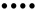) to this sample.

However, formation of MeSeH from a mixture of MSeA and GSH has never been precisely quantified. Because of its volatility and reactivity, MeSeH is difficult to detect and its identification usually relies on indirect methods. To study the above reactions, we first used a spectrophotometric method. UV-visible spectra were recorded after mixing 250 µM MSeA with increasing amounts of GSH. As shown in Figure 1A, a peak at 260 nm appeared in the spectra immediately after addition of GSH. Then, spectra remained stable over time. The intensity of this peak increased with increasing GSH concentrations until a molar GSH:MSeA ratio of 3:1 was reached. Further increase of this ratio did not result in significant modifications, apart from a slight decrease of the peak intensity. This stoichiometry suggests a reduction of MSeA by a three-fold amount of GSH yielding the selenylsulfide MeSeSG and GSSG, according to Equations (1) and (2). Significant production of MeSeH by Equation (3) would have consumed additional GSH molecules and would have resulted in a stoichiometry higher than 3. Analysis of the reaction products between MSeA and a 10-fold excess of GSH by electrospray mass spectrometry detected compounds at *m*/*z* = 307, 613 and 402, corresponding to GSH, GSSG and MeSeSG, respectively. Formation of MeSeSG is in accordance with a study by Kice and Lee [31] showing that benzeneseleninic acid reacts with excess thiols with a stoichiometry of three thiols per seleninic acid to give the selenenylsulfide. However, we cannot exclude at this stage that some MeSeH was formed and reacted with MeSeSG producing GSH and DMDSe (Equation (4)), which is not detectable by electrospray mass spectrometry.

(4) MeSeH + MeSeSG ↔ DMDSe + GSH

If such was the case, a stoichiometry of three thiols per seleninic acid would still be observed:

$$2\;\mathrm{MSeA}\;+\;6\;\mathrm{GSH}\;↔\;\mathrm{DMDSe}\;+\;3\;\mathrm{GSSG}\;+\;4\;H_{2}O$$

To test this possibility, we prepared MeSeSG and studied the exchange reactions (3) and (4) using spectrophotometry. Figure 1B shows the absorption spectra of the different selenium compounds involved in these reactions. Methylselenol, obtained upon reduction of MSeA by TCEP, is characterized by a peak at 252 nm, typical of aliphatic selenolates. MeSeSG and DMDSe, the oxidized form of MeSeH, both display weak absorption above 240 nm with broad peaks centered around 275 and 310 nm, respectively.

We first studied the reaction of MeSeH with MeSeSG. Figure 1C shows the spectrum of MeSeH, produced by reduction of DMDSe with a stoichiometric amount of TCEP. Upon addition of an equimolar concentration of MeSeSG, the signal at 252 nm disappeared immediately (within the time necessary to record the spectrum) and was replaced by a spectrum characteristic of DMDSe, suggesting that MeSeH reacts rapidly with the selenylsulfide to form the diselenide and that the equilibrium of reaction (4) is in favor of the latter.

Next, we studied the reaction of DMDSe with GSH. Because the equilibrium of reaction (4) is shifted towards the right, a large excess of GSH had to be used to obtain a detectable signal change at 252 nm. 100 or 200 µM DMDSe was mixed with 1 to 5 mM GSH and the absorbance at 252 nm was recorded as a function of time.

**Figure 2:**
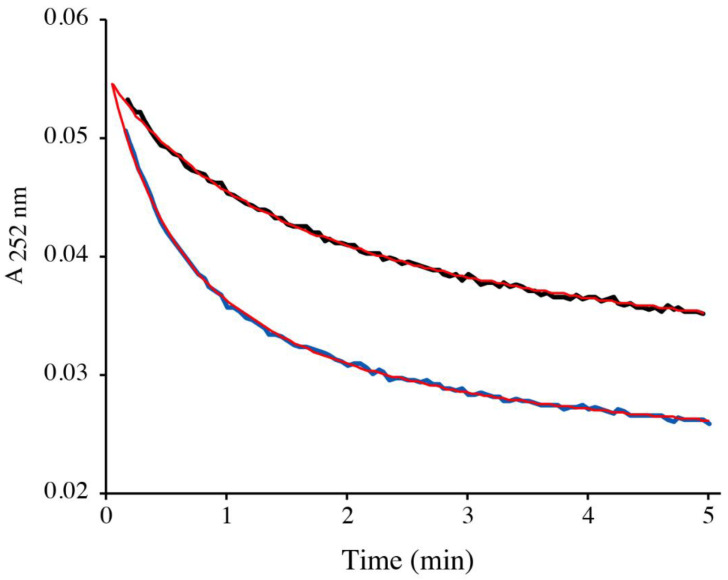
Reduction of DMDSe by GSH. 100 µM DMDSe was mixed with 1 mM GSH (containing 15 µM GSSG) in the absence (black trace) or presence (blue trace) of 50 µM added GSSG in 100 mM potassium phosphate, pH 7.4. The absorbance at 252 nm was recorded as a function of time starting 7 to 8 s after mixing. The absorbance of a solution of 100 µM DMDSe alone, measured with a control sample, was 0.016. The experimental data were fitted to the kinetic model described in Supplementary Materials (red lines).

Figure 2 shows the kinetic traces for the reduction of 100 µM DMDSe by 1 mM GSH. The first experimental point was recorded 7 to 8 s after mixing. The change in absorbance during this dead time suggested that DMDSe rapidly reacted with GSH to form MeSeH. This burst was followed by a slow decrease of the absorbance at 252 nm, which we interpreted as the reaction of MeSeH with the GSSG contaminating the GSH solution. To confirm this hypothesis, we reproduced the experiment after the addition of 50 µM GSSG to the reaction mixture. In agreement with our interpretation, the initial bursts in absorbance were independent of the presence of additional GSSG whereas the initial velocity and the amplitude of the slow phase of the reaction were increased when GSSG concentration was increased (Figure 2). The experimental curves were fitted according to a kinetic model describing Equations (5) and (6) (see Supplementary Materials). Because both forward and backward reactions of Equation (6) are rapid as shown above, we assumed that equilibrium of this equation was achieved at all times. In this model, changes in absorbance, therefore, depend on k_1_ and k_−1_, the rate constants for Equation (5), and K2, the equilibrium constant of Equation (6).

(5) $$\mathrm{MeSeSG}\;+\;\mathrm{GSH}\begin{array}{c} k_{-1}\\ ⇆\\ k_{1} \end{array}\;\mathrm{MeSeH}\;+\;\mathrm{GSSG}\;\;(K1=\;k_{1}/\;k{-}_{1})$$

(6) $$\mathrm{MeSeH}\;+\;\mathrm{MeSeSG}\begin{array}{c} k_{-2}\\ ⇆\\ k_{2} \end{array}\;\mathrm{DMDSe}\;+\;\mathrm{GSH}\;(K2=\;k_{2}/\;k{-}_{2})$$

The best-fit parameter values for the reaction of 100 or 200 µM DMDSe in the presence of varying concentrations of GSH and GSSG are given in Table 1. Mean values and statistical errors calculated from these data are K2 =1067 ± 192, k_−1_ = 325 ± 84 M^−1^ s^−1^, and k_1_ = 0.36 ± 0.14 M^−1^ s^−1^.

To determine more precisely the value of the rate constants, stopped-flow spectrophotometry was employed. To obtain k_−1_, MeSeH was produced by reduction of DMDSe with a substoichiometric amount of TCEP and was immediately mixed with various amounts of GSSG in the stopped-flow instrument. To obtain k_−2_, DMDSe was mixed with GSH. The changes in absorbance at 252 nm were recorded. The kinetic traces from four (for k_−2_) or six different mixtures (for k_−1_) were fitted to the theoretical model. The resulting constants are given in Table 2. The mean value determined for k_−1_ (371 ± 45 M^−1^.s^−1^) was in excellent agreement with the value calculated above. A value of (3.7 ± 0.5) 10^4^ M^−1^.s^−1^ was calculated for k_−2_. We could not obtain an accurate value for k_2_ because the reaction between MeSeH and MeSeSG was too fast to be precisely quantified. However, an estimated value of k_2_ close to 4 10^7^ M^−1^.s^−1^ could be calculated from the values of K2 and k_−2_.

**Figure 3:**
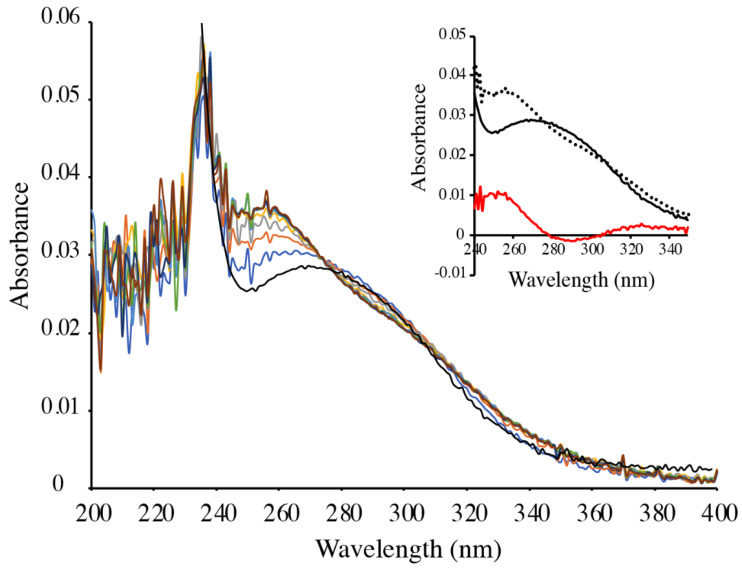
Reduction of MeSeSG by GSH. 100 µM MeSeSG was mixed with 10 mM GSH (containing 150 µM GSSG) in 100 mM potassium phosphate, pH 7.4, and UV absorption spectra were recorded after 20 s (blue line); 60 s (orange); 100 s (grey); 140 s (yellow); 180 s (light blue); 220 s (green); 260 s (dark blue); 300 s (brown). The spectrum of a solution of 100 µM MeSeSG alone is displayed in black. Inset: The spectrum of MeSeSG (black solid line) was subtracted from that of the products of the reaction after 5 min (dotted line) to obtain the difference spectrum (red line).

The selenol/diselenide exchange reactions (5) and (6) were also studied starting from MeSeSG and GSH. 100 µM MeSeSG was mixed with 10 mM GSH and UV-spectra (200–400 nm) were recorded as a function of time (Figure 3). The increase in absorbance at 252 nm indicates that methylselenol was slowly formed and that ~2.2 µM MeSeH was produced when equilibrium was reached. Changes in absorbance observed at 290 nm and 340 nm (Figure 3, inset) indicate that DMDSe was also formed. The kinetic traces at 252 and 340 nm were simultaneously fitted to the kinetic equation system corresponding to Equations (5) and (6). The constants calculated from three independent experiments (K2 = 692 ± 140, k_−1_ = 339 ± 195 M^−1^.s^−1^ and k_1_ = 0.27 ± 0.05 M^− 1^.s^−1^) are in good agreement with those determined above.

The equilibrium constants for thiol/diselenide and selenol/disulfide exchange reactions determined in this study (K1 ~7.3 10^−4^; K2 ~ 1000) are comparable to those determined for cysteine/selenocysteine in [32]. In addition, our results confirm that the reaction of selenols with selenylsulfides are several orders of magnitude faster than with disulfides [32].

**Figure 4:**
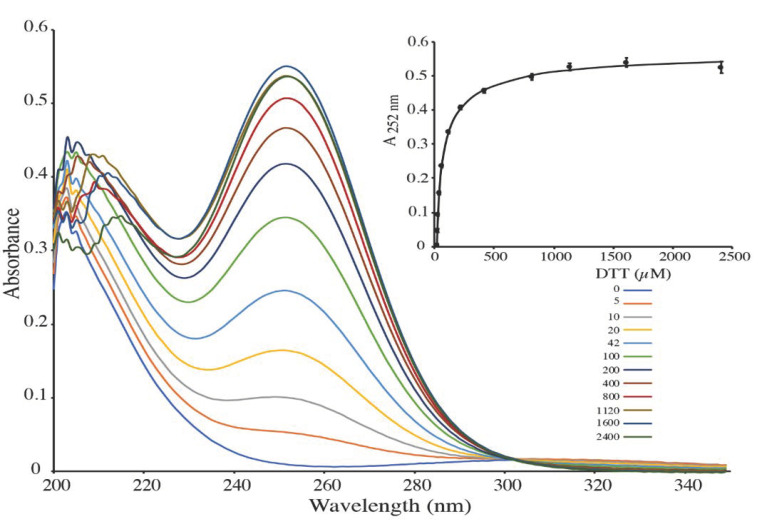
Redox titration of DMDSe with DTT. 50 µM DMDSe was mixed with increasing concentrations of DTT (0 to 2400 µM) in 100 mM potassium phosphate, pH 7.4 and UV absorption spectra were recorded. Inset: The absorbance at 252 nm was plotted against DTT concentration and fitted to the theoretical equation.

To further validate our model, we performed a redox titration of DMDSe with DTT. DMDSe (approx. 50 µM) was mixed with increasing concentrations of DTT (0 to 2500 µM) and UV-spectra (200–400 nm) were recorded (Figure 4). The absorbance of the samples at 252 nm was plotted as a function of the DTT concentration added to the sample (Figure 4, inset). A value for the apparent equilibrium constant Keq ([DTT_ox_][MeSeH]^2^)/([DTT][DMDSe]) of 1.15 ± 0.12 10^−4^ M was estimated by fitting experimental values from two independent experiments to the equation described in Supplementary Materials. From the known value of the equilibrium constant of the reaction between GSH and DTT ([GSH]^2^·DTT_ox_]/([GSSG]·[DTT]) = 200 M [33,34]), we calculated that the equilibrium constant between DMDSe and GSH (K_DMDSe/GSH_ = K1/K2 = [GSSG]·[MeSeH]^2^/([DMDSe]·[GSH]^2^) was equal to 5.8 ± 0.6 10^−7^. This value agrees well with the value of K1/K2 = 7.3 10^−7^ calculated from the above kinetic experiments. From the value of K_DMDSe/GSH_ and the redox potential of the GSSG/GSH couple E°’(GSSG/GSH) = −264 mV at pH 7.4 [35], a value of −446 ± 2 mV can be calculated for the redox potential of the DMDSe/MeSeH couple.

**Figure 5:**
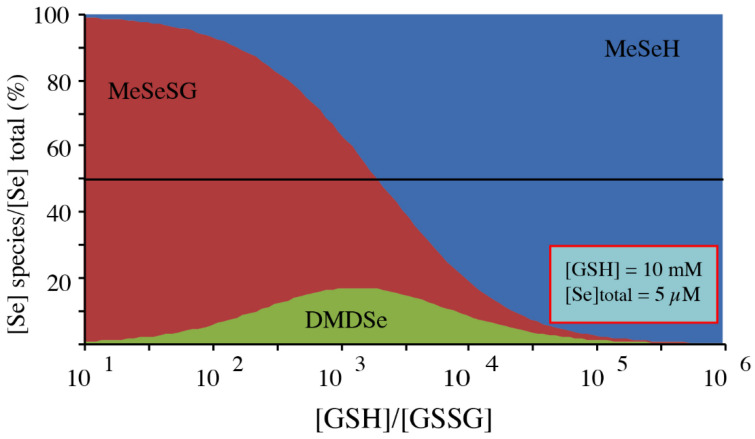
Equilibrium distribution of MeSeH, MeSeSG, and DMDSe as a function of the GSH/GSSG ratio for a GSH concentration of 10 mM and a total Se concentration of 5 µM ([total Se] = [MeSeH] + [MeSeSG] + 2 [DMDSe]). The curves were obtained using values of 1350 for 1/K1 and 1000 for K2.

This value is lower than the redox potentials reported for other unconstrained diselenide bonds such as that of selenoglutathione (−407 mV [36]) or selenocystine (−386 mV at pH 7.0 [37]) and indicates that the reduction of DMDSe requires strong reducing conditions. However, glutathione is abundant in the eukaryotic cytosol with an estimated concentration of 10 mM in *S. cerevisiae* [38]. Moreover, cytosolic GSSG concentration is tightly regulated and the GSH/GSSG ratio was estimated to be as high as 50,000:1 in the yeast cytosol [39]. From the equilibrium constants calculated above, we can predict the equilibrium distribution of MeSeH, MeSeSG, and DMDSe concentrations (Figure 5). For GSH/GSSG ratios > 5000, MeSeH is the major Se-species at total Se concentrations of 1–10 µM. Therefore, it can be expected that, in eukaryotic cells, GSH is able to efficiently reduce both DMDSe and MeSeSG into MeSeH. In contrast, in the in vitro experiments shown in Figure 1, reduction of MSeA by GSH generated GSSG. Therefore, the GSH/GSSG ratio was low and MeSeH is not expected to be significantly produced in these conditions. For example, according to our equilibrium constants values, reaction of 250 µM MSeA with a 10-fold excess of GSH is expected to produce 150 µM MeSeSG, 50 µM DMDSe, and only 0.58 µM MeSeH.

### 2.2. Enzymatic Reduction by Glutathione Reductase and Thioredoxin Reductase

**Figure 6:**
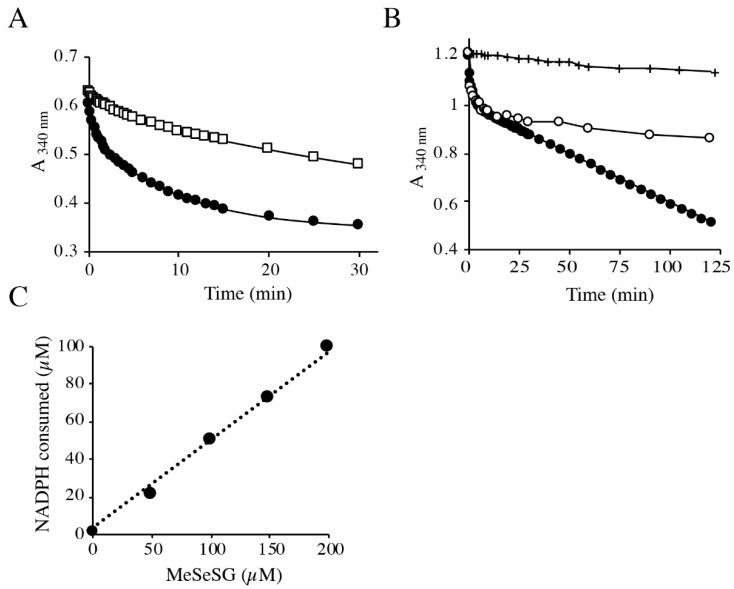
Reduction of MeSeSG or DMDSe by *S. cerevisiae* glutathione reductase. (**A**) NADPH-dependent reduction of MeSeSG (☐) or GSSG (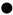). The reaction mixtures contained 100 µM NADPH, and 50 µM of the substrate under study in 100 mM potassium phosphate, pH 7.4. The reactions were started by adding 1.5 nM GR to the GSSG mixture or 73 nM GR to the MeSeSG mixture. (**B**) Aerobic (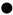) and anaerobic (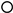, +) consumption of NADPH in the presence of GR and 100 µM DMDSe (+) or 100 µM MeSeSG (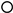, 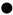). The reaction mixtures contained 200 µM NADPH, 100 nM GR in 100 mM potassium phosphate, pH 7.4, at 22 °C. Anaerobic reactions were performed in a glove box under nitrogen atmosphere. (**C**) Stoichiometry of NADPH consumption as a function of MeSeSG concentration in anaerobic conditions. NADPH consumption was followed in anaerobic conditions with 0 to 200 µM MeSeSG added to the reaction mixture as in B). The amount of NADPH consumed after 125 min was calculated from the ∆A_340_ after subtraction of the value in the absence of substrate.

To determine how MeSeH could be, alternatively, produced in vivo, we tested whether MeSeSG is a substrate for the NADPH-dependent glutathione reductase (GR). Although GR is known to be very specific for GSSG, Ganther [40] has shown that selenodiglutathione (GSSeSG) was a good substrate of GR from yeast. The reduction reaction was followed by measuring NADPH oxidation at 340 nm. In Figure 6A, the reduction rate of MeSeSG was compared to that of GSSG. In our experimental conditions (100 mM potassium phosphate, pH 7.4, 22 °C), 1.5 nM of *S. cerevisiae* GR reduced 35 µM of GSSG in less than 15 min. The change in the absorbance at 340 nm was weak when MeSeSG (50 µM) was used as substrate in the same conditions, but became significant when the concentration of GR in the assay was increased to 73 nM. Initial velocities of NADPH oxidation, deduced from the slope of the absorbance variation at 340 nm during the first min of the reaction, were 7.5 and 0.08 µmol NADPH oxidized min^−1^ nmol^−1^ for GSSG and MeSeSG, respectively.

These results show that MeSeSG acts as a substrate for GR, albeit at a rate reduced nearly 100-fold compared to GSSG. Upon addition of enzyme to MeSeSG, there was a rapid phase of NADPH oxidation followed by a slower continuous oxidation phase (Figure 6B), suggesting that a product of the reaction may redox-cycle with oxygen. To confirm this hypothesis, experiments were performed in anaerobic conditions. The initial phases of the reaction were identical in aerobic and anaerobic conditions, whereas the slow phase of NADPH oxidation was oxygen-dependent (Figure 6B). As shown in Figure 6C, 0.52 mol of NADPH was oxidized per mol of MeSeSG at the end of the experiment in anaerobic conditions. This stoichiometry can be explained by the reaction between the MeSeH produced by the GR-catalyzed reaction (7) with MeSeSG to produce DMDSe (reaction 4). Because the equilibrium constant of Equation (4) is high, the reverse equation of this equation is negligible until almost all the initial MeSeSG is consumed. In these conditions, Reactions (7) and (4) result in the overall reaction (8).

(7) $$\mathrm{MeSeSG}+\;\mathrm{NADPH}\;+\;H^{+}\;\to \;\mathrm{MeSeH}\;+\;\mathrm{GSH}\;+\;{\mathrm{NADP}}^{+}$$

(8) $$2\;\mathrm{MeSeSG}+\;\mathrm{NADPH}\;+\;H^{+}\;\to \;\mathrm{DMDSe}\;+\;2\;\mathrm{GSH}\;+\;{\mathrm{NADP}}^{+}$$

The sub-stoichiometric NADPH consumption in the absence of dioxygen implies that the DMDSe produced in this reaction is not reduced by GR. As expected, when 100 µM DMDSe was mixed with 100 nM GR in anaerobic conditions, the consumption of NADPH was lower than 0.1 µM/min, confirming that DMDSe is not a substrate of GR (Figure 6B).

An oxygen-dependent non-stoichiometric consumption of NADPH was previously reported in the reaction between selenite or selenodiglutathione and the thioredoxin system [41,42]. The authors proposed that the selenolates produced in the reaction continuously react with oxygen to catalyze the oxidation of TRX. In the present case, the non-stoichiometric consumption of NADPH may similarly be explained as follows: the reaction with oxygen of MeSeH formed in reaction (7) will produce DMDSe, which will react with GSH (reaction 4), regenerating MeSeSG. MeSeSG will again be reduced by GR consuming NADPH and the reaction will proceed until all the NADPH is exhausted.

**Figure 7:**
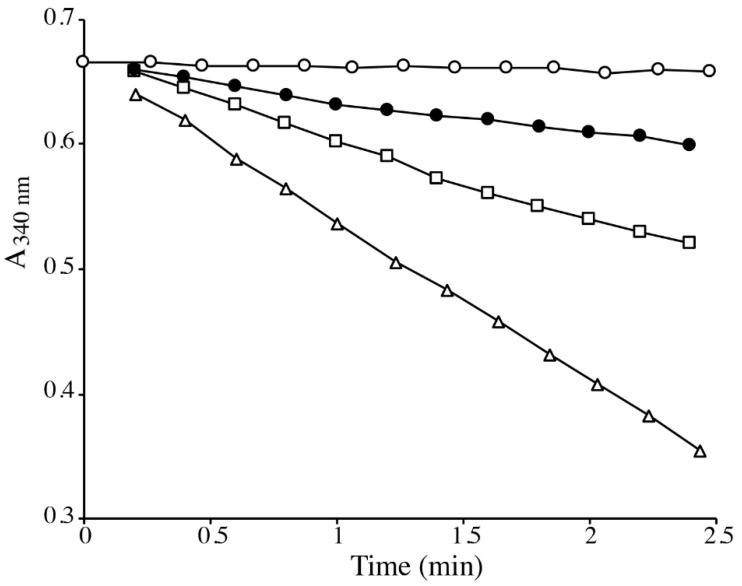
Reduction of MeSeH precursors by the TRX/TRR system. The reaction mixtures contained 100 µM NADPH, 2 µM TRX and 0.5 µM TRR from *S. cerevisiae* in 100 mM potassium phosphate, pH 7.4, 22 °C. The reactions were started by adding buffer (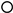), or 50 µM of DMDSe (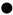), MeSeSG(☐), or MSeA(∆).

The main intracellular disulfide reductase responsible for maintaining proteins in their reduced state is thioredoxin (TRX), which is reduced by electrons from NADPH via thioredoxin reductase (TRR) [43]. The TRX/TRR system acts as a general disulfide reductase with a broad substrate specificity. Several Se-containing compounds, including MSeA, were shown to be directly reduced by the human selenoprotein TRR [44,45]. We studied the reduction of MeSeH precursors (MSeA, MeSeSG, and DMDSe) by a homologous system composed of yeast TRX (2 µM) and TRR (0.5 µM). We first checked NADPH consumption in the absence of TRX. Yeast TRR was unable to directly reduce MeSeH precursors. As shown in Figure 7, TRX/TRR-catalyzed oxidation of NADPH occurred in the presence of 50 µM MSeA, MeSeSG, or DMDSe. DMDSe was the poorest substrate with a reduction rate, in our assay conditions, of 4.6 µM/min, compared to 12 µM/min and 26 µM/min for MeSeSG and MSeA, respectively. Similar rates were observed when the reactions were performed in anaerobic conditions (4.8, 15, and 23 µM/min for DMDSe, MeSeSG, and MSeA, respectively).

### 2.3. Toxicity of MeSeH Precursors

**Figure 8:**
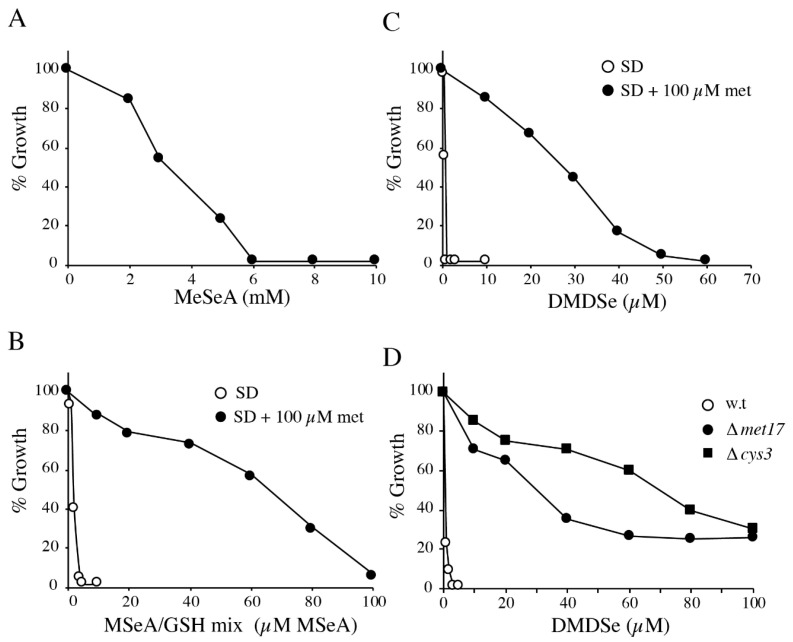
Cell growth inhibition by MeSeH precursors. BY4742 and mutant strains were grown overnight at 30 °C in SD medium or SD medium supplemented as indicated. Exponentially growing cells were diluted to 0.04–0.08 OD_600_ in the same medium and grown for 20–24 h at 30 °C in various concentrations of MeSeH precursors. Growth was monitored by change in OD_600_ and plotted as % of the growth in the absence of toxic. (**A**) BY4742 cells grown in SD medium in the presence of increasing concentrations of MSeA. (**B**) BY4742 cells grown in SD (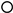) or SD supplemented with 100 µM methionine (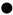) in the presence of increasing concentrations of a mixture containing MSeA with a three-fold excess of GSH. (**C**) BY4742 cells grown in SD (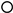) or SD supplemented with 100 µM methionine (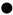) in the presence of increasing concentrations of DMDSe. (**D**) BY4742 (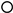) and ∆*met17* (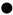), and ∆*cys3* (■) isogenic mutants cells grown in SD medium supplemented with 100 µM cysteine in the presence of increasing concentrations of DMDSe.

The above experiments suggest that MSeA, MeSeSG, and DMDSe are efficiently converted into MeSeH inside the cell and that they are good candidates to study the toxicity of MeSeH. The toxicity of MeSeH precursors was investigated in a growth inhibition assay. Exponentially growing BY4742 cells were diluted to a final optical density of 0.04 OD at 600 nm and incubated in SD medium containing various concentrations of MSeA, MSeA mixed with a three-fold excess of GSH (MSeA/GSH mix), or DMDSe. The cell density of the cultures was measured after 20–24 h. Inhibitory concentration of MSeA was in the mM range. The GI_50_ value (the concentration that reduces by 50% the cell growth at the end of the experiment) for MSeA was 4 mM (Figure 8A). The toxicity of MSeA was exacerbated when it was mixed with a three-fold excess of GSH, producing MeSeSG extracellularly. In this case, the GI_50_ value was around 2 µM (Figure 8B). Likewise, the GI_50_ value of DMDSe was 1 µM (Figure 8C). Because of the high concentration of MSeA needed to inhibit cell growth, we used the MSeA/GSH mix or DMDSe in further experiments. We observed that addition of methionine (100 µM) to the growth medium markedly reduced the toxicity of the MSeA/GSH mix or of DMDSe (Figure 8B,C), the GI_50_ values shifting to 75 and 30 µM, respectively.

This protective effect of methionine prompted us to study the behavior of various mutant strains. A possible explanation for the relief of toxicity by methionine addition could be that the level of intracellular NADPH becomes growth-limiting because reduction of MeSeSG or DMDSe results in continuous oxidation and depletion of NADPH. Because the oxidation of four molecules of NADPH is required to produce methionine from sulfate, an external supply of methionine might help preserving cellular NADPH equivalents and alleviate toxicity. The main source of NADPH is the reaction catalyzed by glucose-6-phosphate dehydrogenase, encoded by *ZWF1*, the first and rate-limiting enzyme in the pentose phosphate pathway. The deletion of *ZWF1* had no effect on the toxicity of the MSeA/GSH mix or DMDSe (result not shown), suggesting that improved growth in the presence of methionine is not related to the preservation of a high NADPH pool. 

Mutants defective in O-acetylhomoserine (OAH)-sulfhydrylase, which catalyzes the incorporation of sulfide into OAH to form homocysteine, were previously selected by resistance to DMDSe [46]. It was speculated that DMDSe was reduced to MeSeH and that the latter was converted into SeMet by OAH-sulfhydrylase, encoded by *MET17*. We have previously shown that SeMet toxicity was mediated by its metabolization to selenocysteine [29]. Therefore, we evaluated the sensitivity to DMDSe of strains deleted for *MET17* or *CYS3*, which encodes cystathionine γ-lyase, an enzyme of the transsulfuration pathway, responsible for cysteine synthesis from methionine. In these experiments, we added 100 µM cysteine as sulfur source. In contrast to methionine, cysteine addition did not affect toxicity. As shown in Figure 8D, both ∆*cys3* and ∆*met17* strains demonstrated a high level of resistance to DMDSe (GI_50_ = 70 µM and 40 µM, respectively). These results suggest that the toxicity of methylated Se compounds are the consequence of their reduction into MeSeH, followed by metabolization of this compound into SeMet by OAH-sulfhydrylase and conversion of SeMet into toxic selenocysteine (SeCys) by the transsulfuration pathway. Thus, the protective effect of methionine against MeSeH toxicity is likely to result from competition between the sulfur- and Se-compounds along the metabolic pathway leading to cysteine, as previously shown in the case of SeMet toxicity [47].

### 2.4. Selenium Content of Cells Exposed to MeSeH Precursors

To determine whether the observed differences in toxicity were related to differences in intracellular Se accumulation, total Se-content was quantified in cells incubated for 30 min with different concentrations of MeSeH precursors. The results shown in Table 3 indicate that exposure to increasing MSeA concentrations resulted in higher total Se-content in the BY4742 strain. When a three-fold excess of GSH was mixed with a non-toxic MSeA concentration (30 µM), cells accumulated 15-times more Se than in the presence of MSeA alone (2331 *vs* 162 µg/g dry weight). Thus, the observed toxicity of a mixture of MSeA and GSH is correlated to an increased Se accumulation, suggesting that MeSeSG is taken up more efficiently than MSeA by yeast cells. Exposure to a toxic concentration of DMDSe also led to a high accumulation of Se.

Total Se- and SeMet-content in the water-soluble fraction of BY4742 or BY4742-∆*met17* cells were compared (Table 4). In the presence of MSeA, ∆*met17* cells accumulated half as much total Se as the parental strain. When ∆*met17* cells were exposed to the MSeA/GSH mix or DMDSe, water-soluble Se-content was reduced more than 25 times and the amount of SeMet was reduced more than 10 times compared to the wild-type. These results suggest that conversion of MeSeH to SeMet by OAH-sulfhydrylase is the major pathway by which MeSeH is converted into selenocompounds that accumulate in the cell.

∆*met17* cells are unable to synthesize methionine in the absence of an organic source of sulfur. However, a small amount of SeMet (around 10 µg/g of cells, which is 4 to 60 times lower than in BY4742) was observed when ∆*met17* cells were incubated with MeSeH precursors. An incorporation of Se in SeMet had already been observed in ∆*met17* cells incubated with selenite [48]. This points to a metabolic pathway resulting in SeMet synthesis independently of OAH-sulfhydrylase. This enzyme belongs to a family of closely related pyridoxal phosphate (PLP)-dependent enzymes that catalyze a wide variety of reactions in amino acid metabolism. In particular, cystathionine beta-synthase (Cys4p), which catalyzes cystathionine synthesis from serine and homocysteine, is structurally homologous to OAH-sulfhydrylase and was shown to possess serine sulfhydrylase activity [49]. It is conceivable that this enzyme has low homoserine sulfhydrylase activity, which would directly generate SeMet from MeSeH. The identification of SeMet in MSeA-treated human lymphoma cells suggests that such a pathway might be relevant in mammalian cells, which cannot synthetize amino acids from inorganic sulfur [50,51].

### 2.5. Exposure to MeSeH Causes Protein Aggregation

**Figure 9:**
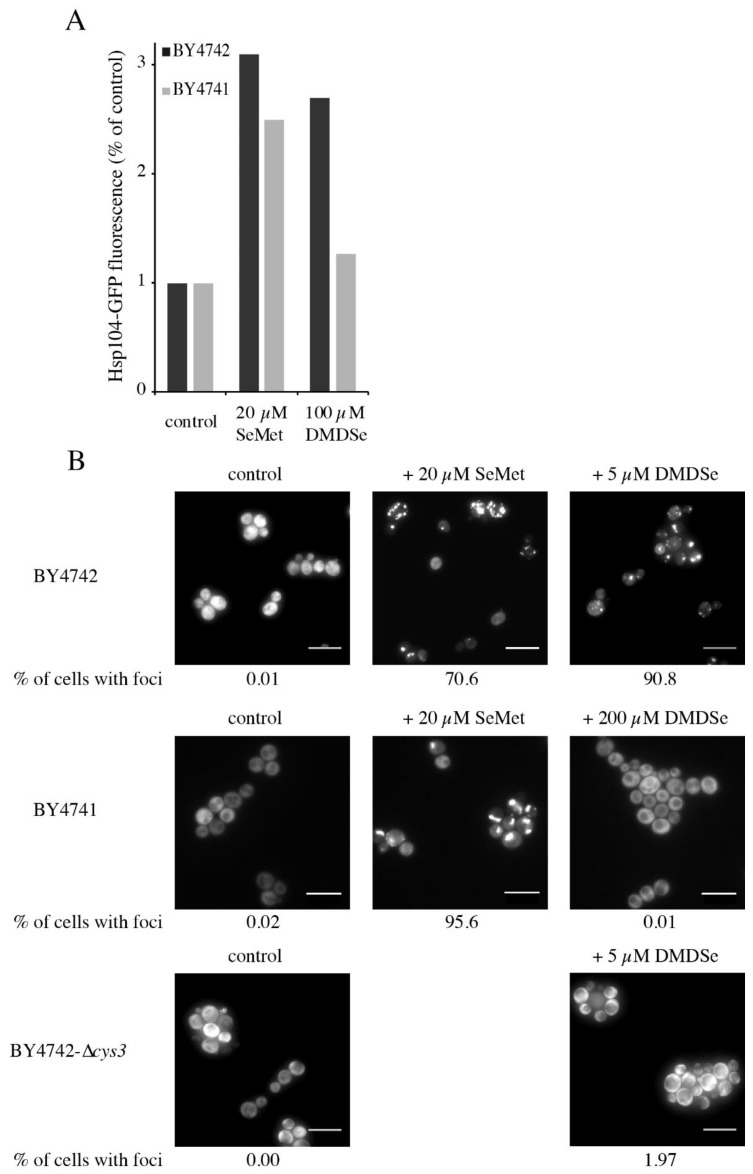
DMDSe promotes protein aggregation in a *MET17*-dependent manner. (**A**) Induction of Hsp104-GFP by SeMet or DMDSe. Exponentially growing BY4742-Hsp104-GFP (dark bars) or BY4741-Hsp104-GFP (light bars) cells were incubated in SC + 100 µM methionine for 2 h at 30 °C in the absence or presence of the indicated concentration of SeMet or DMDSe. The fluorescence in whole cell extracts was recorded at 508 nm and normalized to the optical density of the extracts at 280 nm. The fluorescence intensity in the absence of toxic (control) was set as 1. (**B**) Localization of Hsp104-GFP in cells exposed to SeMet or DMDSe. Hsp104-GFP localization was monitored by fluorescence in living cells after 1 h of exposure to the indicated concentrations of SeMet or DMDSe. BY4742 and BY4742-∆cys3 cells were grown in SD supplemented with 100 µM cysteine, BY4741 cells were grown in SD supplemented with 100 µM methionine. Representative images obtained after maximum intensity z-projection, bar equals 10 µm. The fraction of cells containing at least one Hsp104-GFP focus was determined by manual inspection of 100–200 cells in each condition.

We recently showed that SeMet exposure induced Hsp104 expression and caused an accumulation of toxic protein aggregates in yeast cells resulting from the conversion of SeMet to SeCys by the transsulfuration pathway and the misincorporation of SeCys in the place of cysteine during protein biosynthesis [29]. Protein aggregation was visualized by fluorescence microscopy of live cells expressing an Hsp104-GFP construct. To confirm that MeSeH toxic effects were mediated by its metabolization to SeMet, we compared the effects of DMDSe on the expression (Figure 9A) and localization (Figure 9B) of Hsp104-GFP in BY4742 cells, expressing OAH-sulfhydrylase, to those of BY4741 cells, which are devoid of this activity (*met17*). Exposure to 20 µM SeMet for 2 h induced the expression of Hsp104-GFP in both strains, whereas induction of Hsp104-GFP by DMDSe (100 µM) was dependent on the presence of a functional *MET17* gene (Figure 9A). As shown in Figure 9B, Hsp104-GFP distribution was diffuse in the cytoplasm of unexposed cells, whereas aggregates were visible in BY4742 cells exposed to toxic concentrations of SeMet or DMDSe. At 20 µM SeMet or 5 µM DMDSe, more than 70% of cells contained at least one distinct bright fluorescent focus. In contrast, Hsp104-GFP remained soluble in BY4741 cells exposed to 5 or 200 µM DMDSe. In a previous article, we showed that protein aggregation upon SeMet exposure was suppressed in a ∆*cys3* mutant unable to synthesize SeCys from SeMet [29]. 5 µM DMDSe was unable to induce protein aggregation in a BY4742-∆*cys3* mutant strain. Altogether, these results indicate that intracellular formation of MeSeH leads to toxic protein aggregation in wild-type yeast cells.

## 3. Conclusions

Our results show that MeSeH can be efficiently produced from MSeA, MeSeSG and DMDSe, provided that the cellular GSH/GSSG ratio is sufficiently high. Enzymatic reduction of MeSeH precursors could also contribute to MeSeH formation in vivo. From toxicity experiments with MeSeH precursors, we conclude that MeSeH is toxic for yeast cells and that this toxicity results from (i) conversion of MeSeH into SeMet by OAH- sulfhydrylase, (ii) conversion of SeMet into SeCys by the transsulfuration pathway and (iii) SeCys misincorporation during protein synthesis, which produces toxic protein aggregates. Because humans can synthetize only very low levels of SeMet from MSeA [50,51], this pathway is not likely to account for MSeA anti-cancer effects. However, at higher concentrations, MeSeH is toxic to *met17* yeast cells which, like human cells, lack OAH-sulfhydrylase activity. We are currently studying the mechanism of toxicity of MeSeH precursors in a methionine-auxotroph yeast strain.

## 4. Materials and Methods

### 4.1. Reagents and Enzymes

MSeA was purchased from Aldrich (Saint-Louis, MO, USA); GSH, GSSG, NADPH, DTT, DMDSe, and TCEP from Sigma (Saint-Louis, MO, USA). DMDSe was prepared as a 20 mM stock solution in 100% ethanol stored at −20 °C. Glutathione reductase from baker’s yeast (205 units/mg, 2.2 mg/mL) was from Sigma (Saint-Louis, MO, USA). Yeast thioredoxin (9.7 units/mg, 88.5 µM) was from Fisher Scientific (Waltham, MA, USA). Yeast thioredoxin reductase (25.6 units/mg) was purchased from Abnova (Taipei, Taiwan).

### 4.2. Spectrophotometric Measurements

All spectrophotometric measurements were performed with a double beam Agilent Technologies (Santa Clara, CA, USA) Cary 100 spectrophotometer. All experiments were performed in 100 mM potassium phosphate, pH 7.4, at 22 °C. To minimize aerobic oxidation of MeSeH, the buffer was deoxygenized in a glove-box under nitrogen atmosphere until it was used aerobically. During the time necessary to perform the experiments, the rate of oxidation of MeSeH was reduced by 75% compared to that in fully oxygenated buffer. The molar extinction coefficients of MeSeH at 252 nm and 340 nm (4670 and 192 M^−1^ cm^−1^, respectively) were determined from the reactions of MSeA or DMDSe solutions of known concentration with a 10-fold excess of TCEP. Identical results were obtained with MSeA or DMDSe. Calculation of rate and equilibrium constants were made using values of 84, 288, 226, 161, 35 and 107 M^−1^ cm^−1^ for the ε_252nm_ of GSH, GSSG, MeSeSG, DMDSe, DTT, and DTT_ox_ respectively. The values used for ε_340nm_ of GSH, GSSG, MeSeSG, MeSeH, and DMDSe were 3, 5, 15, 192, and 52 M^−1^ cm^−1^, respectively. When reduction reactions with GSH, DTT, or TCEP were analyzed by spectrophotometry, both cuvettes contained the reductant. The reactions were initiated by adding the Se compound in the sample cuvette. Stock solutions of GSH and DTT (100 mM), buffered at pH 7.4, were renewed every day. The concentrations of these stock solutions were determined by Ellman’s assay [52]. The amount of contaminant GSSG (1.2 to 1.5% (mol/mol)) in the GSH solution was measured by reduction with NADPH (200 µM) and glutathione reductase (0.1 units/mL). GSSG concentration was deduced from the change in absorbance at 340 nm, caused by the conversion of NADPH to NADP^+^. DTT solutions contained 0.5 to 0.6% (mol/mol) DTT_ox_, as measured by UV absorbance at 285 nm (ε_285 nm_ = 283 M^−1^ cm^−1^).

### 4.3. MeSeSG Purification

5 mM MSeA and 20 mM GSH were mixed in 100 mM potassium phosphate, pH 7.4, and left to react for 10 min. The mixture was then diluted twice with 20 % (*v*/*v*) acetic acid/water to bring the pH to 3.5 and applied on an Alltima C18 high-performance liquid chromatography column (0.32 × 15 cm, 5 µm), from Grace (Columbia, MD, USA) equilibrated in 0.05% (*v*/*v*) acetic acid/water at a flow rate of 0.4 mL/min. Elution was performed with the same solution for 5 min, followed by a linear gradient from 0.05% acetic acid to 100% methanol in 20 min. The retention times of GSH and GSSG were 3.1 and 11.2 min, respectively. Another peak, likely corresponding to MeSeSG, eluted at 14.8 min. Identity of this peak was confirmed by positive electrospray mass analysis. The result indicated the presence of a major ion species at *m*/*z* = 402.023 with an isotopic distribution in agreement with the presence of a selenium atom. Fractions containing MeSeSG were collected, lyophilized, and aliquoted for storage at −20 °C. After resuspension in 100 mM potassium phosphate, pH 7.4, concentration of MeSeSG was determined from the absorbance of MeSeH at 252 nm after reduction with a ten-fold excess of TCEP.

### 4.4. Stopped-Flow Experiments

An Applied Photophysics (Leatherhead, UK) SX20 and a BioLogic (Willow Hill, PA, USA) SFM-300 stopped-flow apparatus, with cell pathlength of 0.2 and 0.08 cm, respectively, were used to determine kinetic constants. Both instruments had a dead time of 1 ms. All experiments were performed in deoxygenized 100 mM potassium phosphate, pH 7.4, at 22 °C. To determine the reaction rate between MeSeH and GSSG, MeSeH was produced by reduction of DMDSe with a substoichiometric amount of TCEP. Reactions were initiated by mixing equal volumes of MeSeH (final concentrations: 50 or 100 µM) and GSSG (final concentrations: 100, 200, 300, 400, or 600 µM) or equal volumes of DMDSe (final concentrations, 50, 100, or 125 µM) and GSH (final concentrations, 1250, 1667, or 2500 µM). After mixing, the change of absorbance at 252 nm was recorded.

### 4.5. Determination of DMDSe Redox Potential

50 µM DMDSe was mixed with DTT (0 to 2500 µM) in 100 mM potassium phosphate, pH 7.4, at 22 °C. UV-spectra (200–400 nm) were recorded immediately after mixing and after 1 min. Identical results were obtained, showing that chemical equilibrium between reactants was reached. To obtain the equilibrium constant of the reaction, the increase in absorbance at 252 nm as a function of added DTT was fitted to the theoretical equation.

### 4.6. Data Analysis

Experimental data were fitted to theoretical equations described in Supplementary Materials using OriginPro software (OriginLab Corporation, Northampton, MA, USA).

### 4.7. Enzymatic Assays

The reduction reactions were performed at 22 °C in 100 mM potassium phosphate, pH 7.4. When glutathione reductase from *S. cerevisiae* was used, the reaction volume (600 µL) contained 50 or 100 µM of the substrate under study (GSSG, MeSeSG, or DMDSe), 100 to 200 µM NADPH, and glutathione reductase at the indicated concentration. Reduction by the *S. cerevisiae* thioredoxin/thioredoxin reductase system were performed in an assay mixture of 200 µL containing 2 µM thioredoxin and 0.5 µM thioredoxin reductase, 100 µM NADPH, and 50 µM substrates. The oxidation of NADPH was followed at 340 nm using a millimolar extinction coefficient of 6.22 cm^−1^ for NADPH. Anaerobic assays were performed in a glove-box under nitrogen atmosphere.

### 4.8. Strains and Media

The *S. cerevisiae* strains used in this study are derived from strain BY4742 (*MAT*α *his3∆1 leu2∆ lys2∆0 ura3∆0*) or BY4741 (*MAT*a *his3∆1 leu2∆ met17∆0 ura3∆0*). The parental strains and the single mutants were obtained from Euroscarf (Scientific Research and Development GmbH, Oberursel, Germany). The BY4741 strain expressing a GFP-tagged Hsp104 was purchased from ThermoFisher Scientific (Waltham, MA, USA). GFP tagging of Hsp104 in BY4742 was performed by PCR-mediated homologous recombination, and correct integrations were checked by PCR. Standard Synthetic Defined (SD) medium contained 0.67% (*w*/*v*) yeast nitrogen base (Difco, Becton, Dickinson and Company, Sparks, MD, USA), 2% (*w*/*v*) glucose and 50 mg/l of histidine, leucine, lysine, and uracil and was buffered at pH 6.0 by the addition of 50 mM MES-NaOH. Standard Synthetic Complete (SC) medium contained 0.67% (*w*/*v*) yeast nitrogen base (Difco), 2% (*w*/*v*) glucose and 80 mg/l of adenine, uracil and all amino acids (160 mg/mL leucine) except methionine and cysteine and was buffered at pH 6.0 by the addition of 50 mM MES-NaOH. Media were supplemented in methionine and cysteine as indicated in the legends to the figures.

### 4.9. Toxicity Assays

Cells were pre-grown at 30 °C in the medium subsequently used. Exponentially growing cells were inoculated in the same medium to obtain an OD_600_ of 0.04–0.08 and left to grow at 30 °C for 1 h. After the addition of Se compounds, cells were grown at 30 °C and the OD_600_ was recorded after 20–24 h.

### 4.10. Determination of Selenium Content

For the determination of total Se content, BY4742 cells were grown at 30 °C to an OD_600_ of 0.6–0.8 in SD medium, followed by 30 min exposure to the Se-compound under study. For the determination of SeMet and total water soluble Se contents, BY4742 and a ∆*met17* mutant were grown in SD + 100 µM cysteine, followed by 30 min exposure to the Se-compound. Cells were harvested by centrifugation and washed 3 times in an equal volume of ultrapure water. Samples corresponding to 30 OD_600_ units were aliquoted in microfuge tubes and lyophilized. For each condition, at least three aliquots were prepared. The dry weight of each yeast sample was estimated by the difference of weight between the filled and empty microtubes.

Total Se contents were determined in yeast samples after digestion of 4–8 mg (dry weight) of sample with 100 µL of nitric acid and 50 µL of H_2_O_2_. Se concentration in water extracts was determined after extraction of 4–8 mg samples with 200 µL of ultrapure water using ultrasonic bath (Sonorex, Bandelin, Berlin, Germany) for 1 h. After extraction, samples were centrifuged at 20,000× *g* for 15 min. 30 µL aliquots of water extract were used and digested with 100 µL of nitric acid. Digestion was carried out at 65 °C for 4h in a hot block. After digestion, samples were diluted and analyzed by inductively coupled plasma mass spectrometry (ICP-MS) (Agilent 7500cs, Tokyo, Japan) operating in hydrogen collision gas mode (H_2_) to remove possible interferences. Certified reference material (CRM) SELM1 (NRC Canada) was analyzed together with the samples to validate the method and to create a five-points calibration curve.

SeMet concentration was determined after reverse phase HPLC separation on an Acquity UPLC BEH C18 column (150 × 2.1 mm, 1.7 μm), from Waters (Milford, MA, USA). Gradient elution, at a flow rate of 0.35 mL/min, was carried out using eluent A, 0.1% formic acid in water, and eluent B, 0.1% formic acid in acetonitrile. The gradient program was 0–0.5 min 0% B, 10.5–20.5 min up to 25% B, 20.5–27.5 min up to 50% B, 27.5–30 min at 50% B, 30–31 min down to 0% B, 31–35 min 0% B. Samples obtained from water extraction were filtrated through 3kDa cutoff filter and diluted with MilliQ water. A 5 µL aliquot of the supernatant was injected into the reverse phase column each time. SeMet standard (Sigma, Saint-Louis, MO, USA) was used in order to create a calibration curve. ICP-MS detection was achieved using a model 7700 instrument (Agilent) fitted with platinum cones, 1 mm internal diameter injector torch. 7.5% of oxygen was added to plasma gas to avoid carbon deposit on the cones. The RP ICP MS coupling was done via Scott spray chamber cooled to −5 °C [53].

### 4.11. Fluorescence Microscopy

Yeast cells expressing Hsp104-GFP were grown at 30 °C to an OD_650_ of 0.5 in SC medium supplemented with methionine and/or cysteine as indicated in the figure legends, followed by exposure to SeMet or to DMDSe for 1 h. Cells were mounted on glass slides covered with a thin layer of 1% agarose. Differential interference contrast (DIC; Nomarski interference contrast) and fluorescence images were obtained at room temperature using a Zeiss (Oberkochen, Germany) Axio Observer microscope equipped with a 40×, 1.4 NA oil immersion objective. 470 nm excitation at maximum available intensity (4 W cm^−2^) and a filter set 65 HE (EX BP 475/30, BS FT 495, EM BP550/100) were used for fluorescence imaging. The lateral resolution was estimated to be 180 nm. Images were captured with digital camera Zeiss AxioCam MRm. Z-stacks of 15 to 25 images with 260 nm spacing were recorded. Maximum intensity z-projections were performed with ImageJ and the images were analyzed manually.

### 4.12. Fluorescence Spectroscopy

BY4742 or BY4741 cells expressing Hsp104-GFP were grown at 30 °C to an OD_600_ of 0.5 in SC medium + 100 µM methionine, followed by 2 h exposure to 20 µM SeMet or 100 µM DMDSe. Whole cell extracts from 1–3 OD_600_ units were prepared in 50 mM Tris-HCl (pH 7.5), 10 mM MgCl_2_, 250 mM NaCl, 5% (*v*/*v*) glycerol, 10 mM 2-mercaptoethanol, by vortexing cells at 4 °C for 10 × 30 s in the presence of an equal volume of glass beads (500–750 µm). After centrifugation at 10,000× *g* for 10 min, the supernatant was recovered and the optical density at 280 nm was determined. GFP fluorescence was recorded at 508 nm in a Jasco FP-8300 spectrofluorometer using an excitation wavelength of 487 nm (bandwidth 2.5 nm).

## Figures and Tables

**Table 1:** Equilibrium and rate constants deduced from spectrophotometric experiments with mixtures of DMDSe, GSH, and GSSG.

Initial Concentrations (µM)			K2	k_−1_ (M^−1^.s^−1^)	k_1_ (M^−1^.s^−1^)
DMDSe	GSH	GSSG			
100	1000	15 *	1288 ± 7	431 ± 8	0.45 ± 0.02
100	1000	15 * + 50	1184 ± 10	311 ± 4	0.46 ± 0.02
100	2500	37.5 *	812 ± 4	232 ± 3	0.54 ± 0.01
100	5000	75 *	1222 ± 5	226 ± 2	0.15 ± 0.01
200	2500	37.5 *	902 ± 5	388 ± 4	0.30 ± 0.01
200	5000	75 *	994 ± 10	362 ± 5	0.27 ± 0.01

* GSSG present as a contaminant in the GSH solution.

**Table 2:** Rate constants deduced from stopped-flow experiments performed with mixtures of MeSeH and GSSG (left) or GSH and DMDSe (right). Means and standard deviations were calculated from at least three replicate measurements.

MeSeH (µM)	GSSG (µM)	Rate Constant k_−1_ (M^−1^.s^−1^)	GSH (µM)	DMDSe (µM)	Rate Constant k_−2_ (M^−1^.s^−1^)
25	100	418 ± 32	1250	50	2.9 ± 0.7 10^4^
50	200	434 ± 43	1667	67	4.3 ± 1.3 10^4^
50	300	365 ± 70	2500	100	3.6 ± 0.9 10^4^
50	400	351 ± 140	1250	125	3.9 ± 1.3 10^4^
100	400	356 ± 35			
100	600	322± 54			

**Table 3:** Total Se content (µg/g dry weight) of BY4742 cells after 30 min incubation with MSeA, MSeA /GSH mix or DMDSe in SD medium. Means and standard deviations were calculated from three replicate measurements of at least two independent experiments.

Strain	Se Compound Added	Concentration	Total Se (µg/g Dry Weight)
BY4742	MSeA	30 µM	162 ± 32
	MSeA	300 µM	314 ± 25
	MSeA	10 mM	4053 ± 455
	MSeA /GSH mix	30 µM + 90 µM	2331 ± 600
	DMDSe	5 µM	1378 ± 290

**Table 4:** Total Se and SeMet content (µg/g dry weight) in the water-soluble fraction of BY4742 or BY4742-∆*met17* cells grown in SD + 100 µM cysteine and incubated for 30 min in the presence of MSeA, MSeA/GSH mix, or DMDSe. Means and standard deviations were calculated from three replicate measurements of at least two independent experiments.

		BY4742		BY4742-∆*met17*	
Se Compound Added	Concentration	Total Se in Water Fraction	SeMet	Total Se in Water Fraction	SeMet
MSeA	300 µM	118 ± 16	47 ± 11	67 ± 17	12 ± 5
MSeA/GSH mix	30 µM + 90 µM	1462 ± 515	349 ± 159	33 ± 6	6 ± 0.5
DMDSe	5 µM	573 ± 49	81 ± 41	21 ± 5	7.5 ± 3

## Data Availability

Data is contained within the article or supplementary material.

## Supplementary Materials

The following are available online at https://www.mdpi.com/1422-0067/22/5/2241/s1.
