# Supplementary material for: Methylselenol Produced In Vivo from Methylseleninic Acid or Dimethyl Diselenide Induces Toxic Protein Aggregation in Saccharomyces cerevisiae
Source: Int J Mol Sci. 2021 Feb 24;22(5):2241. doi: 10.3390/ijms22052241 (PMC7956261; doi:10.3390/ijms22052241)
Supplement: Supplementary file 1 [file ijms-22-02241-s001.pdf]

Supplementary Materials to

Methylselenol produced in vivo from methylseleninic acid or dimethyl diselenide induces toxic protein aggregation in *Saccharomyces cerevisiae*

Marc Dauplais, Katarzyna Bierla, Coralie Maizeray, Roxane Lestini, Ryszard Lobinski, Pierre Plateau, Joanna Szpunar and Myriam Lazard

Calculation of the equilibrium constant of the reaction between DMDSe and DTT

DMDSe is expected to be reduced by DTT according to the reaction:

$$\mathrm{DMDSe} + \mathrm{DTT} \rightleftarrows 2\ \mathrm{MSeH} + \mathrm{DTT_{ox}}$$

The corresponding equilibrium constant can be written : $K = \frac{[\mathrm{MeSeH}]^2[\mathrm{DTT_{ox}}]}{[\mathrm{DMDSe}][\mathrm{DTT}]}$

The solution of DTT that we used contained 0.5% of oxidized DTT. If c_0 represents the initial concentration of DMDSe, c_1 the initial concentration of total DTT (c_1 = [DTT] + [$\mathrm{DTT_{ox}}$]), α the proportion of $\mathrm{DTT_{ox}}$ in the DTT solution, and ξ the advancement of the reaction (defined as ξ = [MeSeH]/(2.c_0)), the concentrations of the different compounds in the mixture of DMDSe and DTT are:

$[\mathrm{MeSeH}] = 2.\xi.c_0$

$[\mathrm{DMDSe}] = c_0(1- \xi)$

$[\mathrm{DTT}] = (1- \alpha)c_1 - \xi.c_0$

$[\mathrm{DTT_{ox}}] = \alpha.c_1 + \xi.c_0$

Introduction of the these equations in the expression of K yields an equation from which c_1 can be expressed as a function of the other parameters:

$$c_1 = \frac{4\,\xi^3 {c_0}^2 + \xi(1-\xi)c_0.K}{K(1-\alpha)(1-\xi)c_0 - 4.c_0.\alpha.(1-\xi)} \qquad (1)$$

Because DTT was added at the same concentration in the reference and sample cuvettes, the absorbance of the sample at 252 nm recorded by the spectrophotometer was equal to:

$A_{252} = \varepsilon_{\mathrm{MeSeH}}[\mathrm{MeSeH}] + \varepsilon_{\mathrm{DMDSe}}[\mathrm{DMDSe}] + \varepsilon_{\mathrm{DTT}}(\ [\mathrm{DTT}] - (1- \alpha).c_1) + \varepsilon_{\mathrm{DTTox}}(\ [\mathrm{DTT_{ox}}] - \alpha.c_1)$

where ε refers to the molar absorption coefficients of the compounds at 252 nm. Using the above equations giving the concentrations of the different species, we can write the relation linking ξ to the absorbance:

$$\xi = \frac{A_{252} - \varepsilon_{\mathrm{DMDSe}} c_0}{(2\varepsilon_{\mathrm{MSeH}} - \varepsilon_{\mathrm{DMDSe}} - \varepsilon_{\mathrm{DTT}} + \varepsilon_{\mathrm{DTT_{ox}}}).c_0} \qquad (2)$$

The combination of equations (1) and (2) gives an implicit equation relating A_{252} to c_1.The value of K was obtained by fitting this implicit function to the experimental data using OriginPro software.

Calculation of the rate constant for MeSeH aerobic oxidation

MeSeH is readily oxidized by oxygen. To evaluate the rate of this oxidation, we monitored at 22°C the change of absorbance at 252 nm of MeSeH solutions prepared in deoxygenized 100 mM potassium phosphate buffer as described in Materials and Methods. At this wavelength, the absorbance of MeSeH is much higher than that of the oxidation product (DMDSe). Various concentrations of MeSeH were produced by reaction of equimolar concentrations of DMDSe and TCEP (15, 30, 45, 60 μM). The observed rates of decay in the first

10 minutes of the reaction were proportional to the initial concentration of MeSeH. From these results, we deduced that the oxidation of MeSeH was pseudo-first-order:

$$\frac{d[\mathrm{MeSeH}]}{dt} = -k_{ox}[\mathrm{MeSeH}]$$

with a value of k_{ox} experimentally determined equal to 4.2 10^{-4} s^{-1}. For the sake of comparison, we also measured the rate of MeSeH oxidation in a fully oxygenized phosphate buffer. A value of 1.7 10^{-3} s^{-1} was determined.

Kinetic modelling of the reactions between MeSeSG, MSeH, DMDSe, GSH and GSSG

If we consider the system of the two reactions (5) and (6) described in the article :

$$\mathrm{MeSeSG} + \mathrm{GSH} \underset{k_1}{\overset{k_{-1}}{\leftrightarrows}} \mathrm{MeSeH} + \mathrm{GSSG} \quad (5) \qquad \mathrm{MeSeH} + \mathrm{MeSeSG} \underset{k_2}{\overset{k_{-2}}{\leftrightarrows}} \mathrm{DMDSe} + \mathrm{GSH} \quad (6)$$

and the reaction of oxidation of MeSeH, assumed to be first-order with respect to MeSeH with a rate constant k_{ox}, the evolution of the reactant concentrations is governed by the following differential equations :

$$\frac{dy1}{dt} = -k_{ox}.y1 + k_1.y3.y4 - k_{-1}.y1.y5 - k_2.y1.y3 + k_{-2}.y2.y4$$

$$\frac{dy2}{dx} = k_{ox}.\frac{y1}{2} + k_2.y1.y3 - k_{-2}.y2.y4$$

$$\frac{dy3}{dt} = -k_1.y3.y4 + k_{-1}.y1.y5 - k_2.y1.y3 + k_{-2}.y2.y4$$

$$\frac{dy4}{dt} = -k_1.y3.y4 + k_{-1}.y1.y5 + k_2.y1.y3 - k_{-2}.y2.y4$$

$$\frac{dy5}{dt} = k_1.y3.y4 - k_{-1}.y1.y5$$

where y1, y2, y3, y4 and y5 designate the concentrations of [MSeH], [DMDSe], [MeSeSG], [GSH] and [GSSG], respectively.

Absorbance of the sample was introduced as a 6th variable (y6). The value of y6 is given by the formula $(\sum \varepsilon_i\, y_i)$.pl, where ε_i represents the molar absorption coefficient of compound i in $\mu M^{-1}.cm^{-1}$, y_i its concentration in µM and pl represents the path length of the acquisition system expressed in cm. Differentiation of this formula shows that y6 obeys to the equation:

$$\frac{dy6}{dt} = \left(\left(-\varepsilon_1 + \frac{\varepsilon_2}{2}\right).k_{ox}.y1 + (\varepsilon_1 - \varepsilon_3 - \varepsilon_4 + \varepsilon_5)(k_1.y3.y4 - k_{-1}.y1.y5) + (-\varepsilon_1 + \varepsilon_2 - \varepsilon_3 + \varepsilon_4)(k_2.y1.y3 - k_{-2}.y2.y4)\right)pl$$

When the fits were realized at two wavelengths simultaneously (252 and 340 nm), another variable corresponding to the absorbances at the second wavelength was created and another differential equation using the molar absorption coefficients corresponding to this wavelength was implemented.

In all the above equations, k_2 was then replaced by K2.k_{-2}, were K2 designates the equilibrium constant of reaction (6) (K2 = k_2/k_{-2}). The reaction rates for reaction (6) are several orders of magnitude larger than those for reaction (5). Therefore, to analyze experiments aiming at determining the rate constants of reaction (5) (k_1 and k_{-1}), we assumed that reaction (6) was always at equilibrium.

Experimental data were fitted to the solution of this set of 6 differential equations with the application ODE [Ordinary Differential Equation] of Origin using the BDF(Stiff) method. When spectrophotometric experiments with GSH and DMDSe or MeSeSG mixtures were simulated, adjustable parameters were K2, k_1 and k_{-1}. The parameter k_{-2} was fixed at a value of 1 10^5 $M^{-1}.min^{-1}$, large enough to ensure that reaction (6) is permanently close to equilibrium. When stopped-flow experiments with MeSeH and GSSG were analyzed, adjustable parameters were k_{-1} and the initial concentration of MeSeH, fixed parameters were k_{-2} = 1 10^5 $M^{-1}.s^{-1}$, K2 = 1000, k_1 = 0.27 $M^{-1}.s^{-1}$. When stopped-flow experiments with GSH + DMDSe mixtures were analyzed, adjustable parameters were k_{-2} and the initial concentration of DMDSe, fixed parameters were K2 = 1000, k_1 = 0.27 $M^{-1}.s^{-1}$. For the determination of k_{-2}, all the parameters but k_{-2} were fixed : K2 = 1000, k_1 = 0.27 $M^{-1}.s^{-1}$; k_{-1} = 3,71 $\times 10^2$ $M^{-1}.s^{-1}$. All these simulations were performed with k_{ox} = 4.2 10^{-4} s^{-1}. As a control, simulations were also performed with k_{ox} values equal to 0 or 8.4 10^{-4} s^{-1}. The values of the kinetic constants varied less than 10%, using these figures.
